# Schistosome-Derived Molecules as Modulating Actors of the Immune System and Promising Candidates to Treat Autoimmune and Inflammatory Diseases

**DOI:** 10.1155/2016/5267485

**Published:** 2016-08-21

**Authors:** Luis Janssen, Gisele Lorranna Silva Santos, Herick Sampaio Muller, Anderson Rodrigues Araújo Vieira, Tatiana Amabile de Campos, Vicente de Paulo Martins

**Affiliations:** ^1^Departamento de Biologia Celular, Instituto de Ciências Biológicas, Universidade de Brasília, 70910-900 Brasília, DF, Brazil; ^2^Programa de Pós-Graduação em Biologia Molecular, Universidade de Brasília, 70910-900 Brasília, DF, Brazil; ^3^Programa de Pós-Graduação em Medicina Tropical, Universidade de Brasília, 70910-900 Brasília, DF, Brazil; ^4^Programa de Pós-Graduação em Patologia Molecular, Universidade de Brasília, 70910-900 Brasília, DF, Brazil; ^5^Programa de Pós-Graduação em Biologia Microbiana, Universidade de Brasília, 70910-900 Brasília, DF, Brazil

## Abstract

It is long known that some parasite infections are able to modulate specific pathways of host's metabolism and immune responses. This modulation is not only important in order to understand the host-pathogen interactions and to develop treatments against the parasites themselves but also important in the development of treatments against autoimmune and inflammatory diseases. Throughout the life cycle of schistosomes the mammalian hosts are exposed to several biomolecules that are excreted/secreted from the parasite infective stage, named cercariae, from their tegument, present in adult and larval stages, and finally from their eggs. These molecules can induce the activation and modulation of innate and adaptive responses as well as enabling the evasion of the parasite from host defense mechanisms. Immunomodulatory effects of helminth infections and egg molecules are clear, as well as their ability to downregulate proinflammatory cytokines, upregulate anti-inflammatory cytokines, and drive a Th2 type of immune response. We believe that schistosomes can be used as a model to understand the potential applications of helminths and helminth-derived molecules against autoimmune and inflammatory diseases.

## 1. Introduction

Parasitic helminths are able to modulate their host's immune system in order to survive, proliferate, and maintain their population. Their immunomodulation includes polarization to Th2 cell response and promotion of regulatory B lymphocytes (B-regs) and regulatory T lymphocytes (T-regs) [1]. This modulation is not only important in order to understand the host-pathogen interactions and to develop treatments against the parasites themselves but also important in the development of treatments against autoimmune and inflammatory diseases. This idea of application of helminth parasites or their subunits for disease treatment has been drawn from the hygiene hypothesis [2]. The hygiene hypothesis was first proposed by Strachan [3], as he noted that there was an inverse correlation between the occurrence of hay fever in children and the number of siblings they had living in the same house. His observation led to the hypothesis that infections in early childhood helped to adequately develop the regulation of the immune system, whereas the lack of such infections would predispose to the emergence of autoimmune diseases [2].

There are many reviews in the literature regarding the regulation of the immune system by helminths [1, 2, 4]. There are also reviews focused on the role of helminths infection or helminths molecules in controlling allergic diseases [5, 6] and in host's metabolism and inflammatory diseases [7–9]. This protective role not only has been assigned to bias a Th2 immune response but also involves T-regs induction and alternatively activated macrophages (AAM), as well as generation of anti-inflammatory cytokines [7, 10–14].

**Figure 1:**
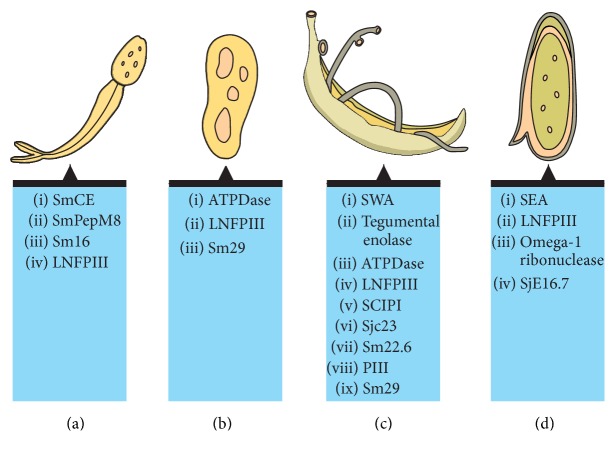
Schistosome-derived molecules from different life stages. Four life stages of* S. mansoni* are represented from (a) to (d): cercariae, schistosomulum, adult male and female pair, and egg. Despite the fact that the egg morphology has been similar to* S. mansoni* egg, the image represents molecules from different schistosome species. Figure created in the free version of “mind the graph” platform: https://mindthegraph.com/, followed by edition using Adobe Photoshop software.

Here, instead of broadening the review with regard to helminths in general, we focus on aspects of the host's metabolism and immune modulation by schistosome-derived molecules, both in terms of its pathogenesis associated with schistosomiasis and in terms of its interaction with the host's immune system in the presence of an autoimmune or inflammatory disease. We believe that schistosomes and schistosome-derived molecules can be used as a model to understand the potential applications of helminths against autoimmune and inflammatory diseases. Later on this paper, in its text, and in Table 1 and Figure 1, we will present some schistosome-derived molecules whose immunoprotective and anti-inflammatory effects have already been demonstrated, as well as other molecules that have the same potential but that still need to be investigated.

## 2. Helminthic Therapy of Autoimmune Diseases: The Clinical Trials and the Potential Importance of Schistosomes

So far, few clinical trials regarding the use of helminths against autoimmune diseases have been done. According to Fleming and Weinstock [45], up until the date of their publication, 28 clinical trials were being conducted, from planning to completion. All of these were using live parasites, instead of parasite-derived molecules or extracts, and the chosen species were* Necator americanus* or* Trichuris suis*. These species have been used in the trials due to their relative safety:* T. suis* causes a zoonotic disease in humans, which is self-controlled, and low inoculum doses of* N. americanus* larvae have proven to be well-tolerated by the human host, despite being able to produce 50 eggs per gram of feces [46, 47]. Those trials which had already finished did not show very promising results in the treatment of autoimmune diseases. Evans and Mitre [46] argue that several factors could be involved in these seemingly negative results, such as the apparent ability of helminths to prevent the development of autoimmune diseases, instead of treating them; the location of helminth infection being incompatible with the desired immunomodulation; the parasitic burden and duration; and the use of concomitant medications and host genetics. Pritchard and colleagues [47] also point out that the absence of heterogeneity of parasitic worm species in the host, during the trials, could have contributed to worse results. Moreover, Fleming and Weinstock [45] claim that host epigenetic background may influence the results and that the process of manufacturing* T. suis* eggs may influence their longevity and vitality. One last factor that could play a role is the parasite species. Although immunomodulation may be a common theme among helminths, each species probably is able to use different strategies to achieve it [46, 47]. Fleming and Weinstock [45] point out that, given the robust effects observed in field studies, there is the possibility that only human pathogens may be properly used in therapy against autoimmune diseases and strict conditions to do so may exist.

Schistosomes, especially* S. mansoni*, can represent a serious threat to human health and thus have not been used in clinical trials up until now. With that in mind, the use of schistosome-derived molecules may represent a viable alternative. Even though there are no clinical trials involving schistosomes as therapy against autoimmune diseases, Nampijja and colleagues [48] report an ongoing interventional field trial in Uganda, which has as one of its goals analysing the effects of anthelminthic treatment on the incidence of allergy-related diseases in communities of Lake Victoria. This study can offer new insights into the applicability of schistosomes and other helminths as treatment against autoimmune diseases, given that half of the local population is infected with* S. mansoni* and the prevalence of other species, such as* Strongyloides stercoralis*,* Trichuris trichiura*, and* N. americanus*, is also high.

## 3. Schistosome Secreted and Surface-Exposed Molecules

The cercaria is the human-infective stage; thus it possesses several adaptations to rapidly invade the host skin. For this penetration to be successful, enzymes are needed to open this barrier [49]. For an efficient, percutaneous infection, glands located at the acetabulum and at the head secrete various substances. In 2006, Curwen and colleagues [50] identified new proteases and immunomodulatory molecules secreted by the cercariae, to adhere to and invade the host skin. Among these molecules, there are three elastase isoforms, which degrade elastin and thus promote the invasion, a metalloprotease (SmPepM8), which is involved in the degradation of the extracellular matrix, and an anti-inflammatory protein, Sm16. The metallopeptidase SmPepM8 belongs to the leishmanolysin family of metalloproteases, responsible for the degradation of the extracellular matrix. This metallopeptidase was the second most abundant component in the secreted proteins by the cercariae [50], an interesting discovery, since this protease class had previously been described only in schistosomula [51].

The Sm16 is another major component of the secreted proteins of the cercariae, which is an important modulator of the innate immune response [52]. Once inside macrophages and after proteolytic degradation, Sm16 stimulates anti-inflammatory signalling in these cells. This signalling seems to be related to the inhibition of cytokine production dependent upon TLR3 and TLR4 signalling, worsening the production of IL-6 and IL-1*α* [52]. Sm16 can also block the activation of IRAK-1 gene, one of the regulating components in the production of the transcription factor NF-*κ*B [53].

Many molecules exposed by the cercariae are able to negatively regulate the host immune response, by several means, such as the induction of IL-10 production and induction of apoptosis of T cells. Among these molecules, one of the most important ones is the glycan Lacto-N-fucopentose-III (LNFPIII) [54]. In 1996, Richter and colleagues [55] administered irradiated cercariae, in order to determine which molecules were the most important in eliciting immune responses. They have found that the levels of antibodies directed towards carbohydrates had risen significantly and that most of these antibodies targeted LNFPIII. The recognition of carbohydrates, especially LNFPIII, by pattern recognition receptors, plays a critical role in triggering a Th2 response [56].

There is strong evidence that glycoconjugates such as glycoproteins and glycolipids from helminths contribute to the immunomodulatory properties during the interaction with host antigen presenting cells [57]. Helminths express a wide variety of glycans linked to proteins or lipids on their surface and on the secretion products. Some glycans have the potential to interact with C-type lectins on dendritic cells, which may directly affect the type of T lymphocyte polarization [58, 59].

During the life cycle of schistosomes in their host, several biomolecules are excreted/secreted from their tegument, present in adult and larval stages, or their eggs and are thus exposed to the host [60, 61]. These molecules can induce the activation and modulation of innate and adaptive responses as well as enabling the evasion of the parasite from host defense mechanisms [61, 62].

The tegument structure of* Schistosoma* spp. is an important interface in the interactions of the parasite with the immune system, but it also participates in other important processes for the fluke, such as nutrition, excretion, signal transduction, and osmoregulation [63–65]. Among the tegument proteins with relevant roles in the interactions with the immune system, there is paramyosin (SCIP-1), found in* S. mansoni* and* S. japonicum*, which is able to selectively bind to the Fc domain of immunoglobulins. This property helps the parasite to evade the immune system, through the adsorption of such molecules [65, 66]. Moreover, it is able to interfere with the complement system, by inhibiting the activity of C9 [67]. Similarly, Sjc23, a protein member of the tetraspanin family and found in the tegument of* S. japonicum*, presents IgG-binding properties [65].

The intravascular presence of adult schistosome may disturb blood flow which would be a potential activator of the blood coagulation, but the formation of these blood clots is not observed around the parasites as evidenced by some studies [68, 69]. Figueiredo and coauthors [70] demonstrated that enolase, tegumental enzyme (Sm-Eno) can promote significant activation of plasminogen. This results in the generation of plasmin, a potent fibrinolytic agent that could be counteracting the formation of blood clots around live worms. The same group demonstrated that another tegumental enzyme, the ectoenzyme SmATPDase1, could be responsible for minimizing the host immune response, inhibiting coagulation of blood and promoting the survival of the parasite through the degradation of ATP and ADP in the blood [71]. The tegumental protein Sm22.6 can inhibit,* in vitro*, the proteolytic activity of thrombin, a central component of the coagulation cascade that converts fibrinogen into fibrin [72, 73]. Thrombin also participates in platelet activation; thereby inhibition of Sm22.6 can decrease thrombin participation in clotting cascade and platelet activation, suggesting a key role for Sm22.6 in pathogen evasion mechanisms [72].

During the egg laying of* Schistosoma*, there is a strong induction of the Th2 response in humans and animal experimental models [60]. This response is caused by soluble egg antigens (SEA) of the parasite [2]. SEA is an agglomerate of proteins and glycoconjugates composed of structural components of the egg embryo along with its secreted products [74]. The omega-1 T2 ribonuclease (*ω*1) is secreted by eggs of* S. mansoni* and is critical in the development of the Th2 response. It is able to bind to the mannose receptor of dendritic cells and is internalized after it. Once internalized, it is able to digest rRNA and mRNA, interfering with the protein synthesis [60]. IPSE/*α*1 is another protein secreted by* S. mansoni* eggs and its secretion leads to the production of IL-4 by basophils, which, in turn, participates in the development of the Th2 response [75]. Lastly, the SjE16.7 protein is also secreted by eggs of* S. mansoni* and is involved in the induction of hepatic inflammation by neutrophil chemotaxis [76].

## 4. Inflammatory and Autoimmune Diseases

Given the capacity of* S. mansoni* to accelerate or abrogate the development of inflammatory and autoimmune diseases, animal models have been created to better understand the molecular mechanisms behind the interactions of* Schistosoma* and such diseases. In the next sections we will present and discuss some of these autoimmune and inflammatory disorders that were associated with amelioration upon schistosome infection or schistosome molecule exposition, such as arthritis, type 1 diabetes (T1D), type 2 diabetes (T2D), metabolic syndrome (MetS), Grave's hyperthyroidism, psoriasis, experimental autoimmune encephalomyelitis (EAE), inflammatory bowel disease (IBD), and airway allergies.

### 4.1. Arthritis

In the case of arthritis, one animal model used is DBA/1 mice that when immunized with bovine type II collagen develop an immune response against their own type II collagen. This model is known as the collagen-induced arthritis (CIA) model. In 2009, Osada and coworkers [15] infected DBA/1 mice with* S. mansoni* cercariae and, after two weeks, the mice were immunized with bovine type II collagen. These mice had their immune response against arthritis compared to uninfected CIA mice. In this study, the researchers found that the infected group had lower arthritis scores and less arthritic paws than the uninfected group and that these characteristics were negatively correlated with the number of parasite pairs found in the mice. They have also found less severe histopathology in the ankles of infected animals, lower anti-type II collagen IgG and IgG2a antibodies in their plasma, and lower production of IFN-*γ*, IL-17, and TNF-*α* and higher production of IL-4 and IL-10 from their spleen cells and lower gene expression of proinflammatory cytokines and Foxp3 in their paws. In 2011, Song and coworkers [16] have found similar results for the* S. japonicum* infection, with the addition of having found lower T cell proliferative responses against type II collagen and elevated Th2 and T-reg spleen cells.

Using a different model, mice that lacked IL-1 receptor antagonist (IL-1Ra), which spontaneously develops autoimmune arthritis, Osada and coworkers [17] showed that infected mice with* S. mansoni* had lower arthritis scores, less severe histopathology, lower production of TNF-*α* and IL-17, and higher production of IL-4 and IL-10. However, they have found higher levels of rheumatoid factor and anti-dsDNA IgG in the infected mice.

While there is evidence for the abrogating effects of* Schistosoma* infection against arthritis, the time required for the infection to reduce the severity of the autoimmune disease is still under debate. Osada and coworkers [15] observed such effects after two weeks of the infection. Song and coworkers [16] noted that the severity of arthritis was aggravated after one week of* S. japonicum* infection. He and coworkers [77] argue that, during the first couple of weeks of infection,* S. mansoni* parasites would not have produced large quantities of eggs, which are necessary for the generation of a Th2 dominating response, responsible for the protective effects against arthritis. From this premise, in their study, the authors have compared the immune response of CIA mice after being infected for different periods of time (2 weeks, 7 to 10 weeks, and 15 weeks). They have shown that immune responses related to protection against arthritis, such as higher production of IL-4, lower production of IFN-*γ*, and decrease in anti-type II collagen IgG production, started to occur on the latter phase of the infection, whereas the 2 weeks' group showed either neutral or faster responses towards the severity of arthritis.

### 4.2. Type I Diabetes

In type I diabetes (T1D), CD8^+^ T cells, CD4^+^ T cells, B lymphocytes, and other plasma cells appear to be involved in the destruction of the pancreatic insulin-producing *β* cells, together with a poor immune regulation [78].

Since* S. mansoni* is able to induce a shift from a Th1 to a Th2 response, which could relieve the autoimmune response; Maron and coworkers [18] have evaluated the cytokine profile and proliferative responses of lymphocytes in nonobese diabetic (NOD) mice after the oral administration of insulin B-chain with or without soluble egg antigens (SEA) of* S. mansoni*. They have observed that both B-chain insulin and SEA administration suppressed the proliferation of lymph node cells, enhanced IL-10 and TGF-*β*, and diminished IL-2 production. They have also observed that the combined administration of SEA and B-chain insulin presented a synergistic effect on IL-10 production and on lymph node cell proliferation.

In 1999, Cooke and coworkers [19] infected 4-5-week-old NOD mice with* S. mansoni*, when the pancreas presents mononuclear cell infiltrations around the pancreatic islets, in an initial physiopathological state of the disease. While the infection did not promote the removal of the infiltrations, mice with lower infection presented high glucose concentrations in blood than the controls. Moreover, infected mice with either adult* S. mansoni* or their eggs presented higher anti-insulin IgM and lower anti-insulin IgG concentrations in sera, which the authors explain to be due to failure of T cell class switching, instead of Th2 response promotion. Lastly, mice that were injected with* S. mansoni* eggs maintained normal glucose concentrations in blood.

Zaccone et al. [20] better characterized how* S. mansoni* could prevent the onset of diabetes in mice. They have shown that four weekly injections of dead worm eggs were sufficient to prevent the onset, as long as they started before the mice reached 5 weeks of age. In addition, SEA or soluble worm antigens (SWA) injected in similar fashion caused fewer mice to have mononuclear infiltrations in the pancreatic islets. Spleen cells transferred from NOD mice that received treatment with eggs or antigens to NOD-SCID mice (NOD mice with impaired T and B lymphocyte development) induced diabetes in at most 50% of the NOD-SCID mice, whereas spleen cells from NOD controls induced diabetes with 100% incidence. The spleen cells from the treated mice produced more IL-5, IL-10, and IL-13 than those from the controls and proliferated more in the presence of the antigens. However, these responses depended on the presence of T lymphocytes amidst the spleen cells, with the exception of IL-10 production, which also depended on B lymphocytes. Moreover, SEA applied with LPS increased the IL-10 production and decreased the IL-12 production of bone-marrow-derived dendritic cells (DCs). Lastly, NOD mice injected with antigens presented more natural killer T (NKT) cells. In addition, Zaccone and coworkers [13] have shown that SEA administration to NOD mice promoted higher levels of T-regs in the pancreas and higher mRNA expression of TGF-*β*, IL-10, IL-4, and IL-35. Spleen cells from NOD mice treated with SEA when depleted of CD25^+^ regained the capacity of diabetes induction in NOD-SCID mice. SEA also favored the generation of T-regs from naïve T CD4^+^ cells and directly inhibited the proliferation of CD4^+^ cells and induced their expression of TGF-*β* and galectins. Cooke and coworkers [21] expanded their studies on the macrophage and DCs response. They were able to demonstrate that DCs incubated* in vitro* with SEA express more mRNA of lectins, such as galectins, produce more TGF-*β*, and express fewer mRNA of IL-12p35 and IL-12p40. Macrophages isolated from NOD mice stimulated with SEA expressed more mRNA related to IL-2, IL-6, IL-10, and TGF-*β*. They have also observed increase in the expression of arginase-1 and arginase-2, Fizz-1, galectin-3, and the mannose receptor, which, together with TGF-*β*, indicate that these macrophages were alternatively activated.

### 4.3. Type 2 Diabetes

Although type 2 diabetes (T2D) is not an autoimmune disease* per se*, immunological factors are involved in the predisposition to develop the disease, such as the obesity-induced low-grade chronic inflammation [79]. T2D is closely related to metabolic syndrome (MetS) which will be discussed in the sections below.

Using high-fat diet-induced obese C57BL/6 mice, Hussaarts and coworkers [22] have shown that SEA administration to these mice promoted eosinophils and Th2 associated cells in their white adipose tissue and hepatic tissue, along with alternatively activated macrophage (AAM) polarization in the white adipose tissue.

Bhargava and coauthors [23] demonstrated that administration of Lacto-N-fucopentose-III (LNFPIII), a LewisX 4 containing immunomodulatory glycan found in human milk and on parasitic helminths, improved glucose tolerance and insulin sensitivity in diet-induced obese mice model. Authors argued that it was partly mediated through increased IL-10 production by AAM and dendritic cells, leading to reduced white adipose tissue inflammation and increased response of adipocytes to insulin.

### 4.4. Psoriasis

The immunomodulatory effect of LNFPIII was also evidenced by Atochina and Harn [27] showing that fsn/fsn mutant mice, models for the study of psoriasis, did not develop skin lesions after the application of two doses of LNFPIII. Moreover, they have observed F4/80^+^ cell levels and CD4^+^/CD8^+^ ratios in their lymph nodes to be more similar to healthy mice after the application of this glycan. The Th2 polarized immune response, induced by LNFPIII, was attributed as a possible reason for the amelioration of skin lesions.

### 4.5. Graves' Hyperthyroidism

Nagayama et al. [26] injected thyroid-stimulating hormone receptor in mice as a means to model Graves' hyperthyroidism and evaluate the potential modulatory effects of this disease by* S. mansoni*. Their results indicate that mice infected by* S. mansoni* before the ad-TSHR injections lead to lower T_4_ production (a thyroid hormone), lower anti-TSHR IgG2 levels, higher production of IL-10, and impaired production of IFN-*γ* upon exposure of TSHR by their spleen cells. Moreover, mice treated with SEA (soluble egg antigens), instead of having been infected, also presented lower T_4_ production than SWA (soluble worm antigens) exposed mice or control mice. These results indicate that the effective molecules were, possibly, egg-derived molecules.

### 4.6. Metabolic Syndrome

Recently, increasing evidence is arising that supports the association of helminths infections, or helminths derived molecules, with prevention and lower prevalence of metabolic syndrome [9]. In 2013 a cross-sectional study in a rural area of China demonstrated that individuals with previous* S. japonicum* infections had lower diabetes prevalence (14.9% versus 25.4%) and lower metabolic syndrome (14.0% versus 35.0%) compared with uninfected persons [80]. In this study, patients previously infected with schistosome also had lower levels of adjusted fasting blood glucose, postprandial blood glucose, glycated hemoglobin A1c (HbA1c), insulin resistance, triglycerides (TGs), and low density lipoprotein cholesterol (LDL-C) [80].

Dyslipidemia (abnormal cholesterol levels in blood) and inflammation of the white adipose tissue, two lipid disorders in metabolic syndrome, have been correlated to inflammatory biomarkers such as cytokines TNF-*α* [81, 82] and IL-6 [83] and the C-reactive protein [83]. This alteration in blood lipid levels coupled to inflammatory milieu is implicated with obesity-induced inflammation, insulin resistance (T2D), and metabolic dysfunction [8, 84–86]. Thus, the ability of helminths to induce anti-inflammatory cytokines biasing to a Th2 immune response seems to be the mechanism behind the preventive effect of schistosome molecules [10–13].

Two animal models have been used to explore the lipid metabolism aspect in metabolic syndrome and the role of helminth interaction, the first one is apolipoprotein E deficient (apoE^−/−^) mice and the second one is low density lipoprotein receptor deficient (LDLR^−/−^) mice. ApoE and LDLR deficient mice develop atherosclerosis in a short time, which would be difficult to achieve in nondeficient mice [87]. Stanley and coworkers [24], using apoE^−/−^ mice, showed that* S. mansoni* eggs, but not the adult worms, were responsible for the molecules acting on serum and liver cholesterol and lipid reduction. They also demonstrated that single sex worm infections did not have the same outcome regarding blood and liver lipid reduction [24]. Using LDLR^−/−^ mice Wolfs and colleagues [25] verified the effect of SEA to successfully reduce serum cholesterol levels and systemic inflammation.

### 4.7. Autoimmune Encephalomyelitis

Experimental autoimmune encephalomyelitis (EAE) is an animal model for the study of multiple sclerosis, in which there is chronic inflammatory demyelination of mice central nervous system (CNS). Studies conducted in this experimental model have demonstrated a marked inverse correlation between the occurrence of helminthic infection and the development of this type of autoimmune disease [30, 31]. Sewell and colleagues [30] in 2003 showed that mice, immunized with* S. mansoni* eggs, had reduced severity of EAE. The elimination of the disease has been associated with immune deviation in the periphery and in the CNS, which was demonstrated by the decrease in IFN-*γ* levels and an increase in IL-4, TGF-*β*, and IL-10 levels in the peripheral system and increased IL-4 and T cell infiltrate in the brain. In another study, La Flamme and coauthors [31] have demonstrated that the cellular composition of the spinal cord and the brains of mice infected with* S. mansoni* showed decreased inflammation of the central nervous system (CNS), particularly a reduction of macrophages and CD4^+^ T cells. The authors argue that these findings suggest that schistosomiasis can downregulate the onset of EAE by downregulating the production of proinflammatory cytokines reducing inflammation of the CNS.

In 2008, Zheng and colleagues [32] used SEA from* S. japonicum* in EAE, both as a preventive and as a therapeutic treatment against the disease. In order to do so, they have applied SEA both before and after the establishment of EAE in C57BL/6 mice. In mice preimmunized with SEA, the onset of EAE could not be delayed, but the severity of the disease was reduced. However, both delay and reduction of EAE severity were observed when SEA was used after EAE was induced. Cultures of splenocytes isolated from mice and restimulated produced more IL-4 and less IFN-*γ* if they came from SEA immunized mice and this cytokine expression pattern was also observed by quantitative PCR (q-PCR). Interestingly, when they assayed the cytokine profile of mouse sera, they did not observe differences in cytokines between preimmunized and control groups.* In vivo* cytokine expression experiments showed only a difference in IL-4 expression levels in spinal cords between preimmunized and control groups, via q-PCR. At last, preimmunized mice presented less demyelination and inflammation in spinal cords.

In 2012, Zhu and colleagues [33] used LNFPIII as a therapy for EAE. Mice treated with the carbohydrate had EAE with reduced severity, though the onset could not be delayed. They have also observed less CNS inflammation in the treated mice. Splenocytes isolated from LNFPIII-treated mice, after restimulation, increased the production of IFN-*γ*, IL-4, IL-5, IL-10, and IL-13. It should be noted that even though there was an increased production of IFN-*γ*, there was also an increased production of cytokines commonly associated with a Th2 response. This resulted in a lower IFN-*γ* : Th2 cytokine ratio and thus indicates a Th2 shift in the immune response. Inflammatory monocytes isolated from LNFPIII-treated mice presented higher nitrous oxide production, which suppressed T cells in cultures. These cells also presented higher mRNA expression levels of immune regulation-related enzymes, such as arginase, aldehyde dehydrogenase, and heme-oxidase. They also presented very low expression levels of Th1 related cytokines, such as IL-1*α*, IL-1*β*, IL-6, and IL-12. Finally, bone-marrow-derived DCs migrated less across* in vitro* mouse brain endothelium, as they were treated with increasing amounts of LNFPIII. Afterwards, Kuijk and colleagues [34] have shown that human DCs previously stimulated with LPS or Poly-I : C, after the incubation with* S. mansoni* SEA, have greatly decreased the expression of TNF-*α* and IL-12, when compared to their nonincubated counterparts. They have also observed the increase of OX40L, a ligand related to development of Th2 and T-reg cells, in DCs incubated with SEA or LPS+SEA. These DCs, after incubation with SEA, also promoted Th2 polarization on naïve T cells. After coculturing the incubated DCs with the naïve T cells, a major portion of the T cells started to produce intracellular IL-4, with only a minor part producing INF-*γ*.

### 4.8. Inflammatory Bowel Disease

Inflammatory bowel disease (IBD) is a chronic inflammatory disease of the gastrointestinal tract, including Crohn's disease and ulcerative colitis [88]. Studies suggest that in these autoimmune diseases both genetic factors and also environmental factors contribute to triggering the inflammatory process [89]. Researchers have shown that* S. mansoni* molecules from eggs and the surface of adult worm, such as glycans, possess potent activity to induce Th2 response and anti-inflammatory cytokines such as IL-10 [90, 91]. Ruyssers and colleagues [28] observed that, after injection of adult* S. mansoni* surface proteins in mice that had Trinitrobenzenesulfonic (TNBS) acid induced colitis, there was no significant change in the levels of IFN-*γ* mRNA expression in T lymphocytes isolated from inflamed tissues. However, there was a decrease in IL-17 and IL-5 levels five days after the induction of colitis. In addition, other studies have shown that infection by helminths can modulate the immune system of animals with intestinal disturbances to a state of immunosuppression by the induction of Th2 immune response and suppression of proinflammatory responses Th1 and Th17 and thus suppressing intestinal inflammation [29, 92].

### 4.9. Airway Allergies

The treatment and prevention of airway allergies by schistosomes may be the best characterized application of these worms in the context of inflammatory and autoimmune diseases. The first study in this context was made by Mangan and colleagues [35], in which they have infected mice with either male and female* S. mansoni* cercariae, so that the infection would have the presence of eggs, or male-only cercariae, so that the infection would not have the presence of eggs. After the establishment of acute or chronic infections, the mice were sensitized with ovalbumin (OVA) injections and later challenged via airway, in order to induce airway hyperresponsiveness. The authors observed higher Th2-related cytokine production (IL-4, IL-5, and IL-13) and IL-10 from spleen cells isolated from mice with schistosomes and eggs than from uninfected mice, along with higher IgE production. It should be noted that these cytokines, in this context, actually promote the worsening of the allergy. They have also observed that mice infected with schistosomes and eggs, following allergen challenge, were predisposed to fatal airway allergies, regardless of infection-phase or OVA sensitization. Mice infected with schistosomes and eggs presented higher collagen deposition in their lungs, when compared to uninfected mice. Lastly, IL-13^−/−^ mice infected with schistosomes and eggs in the acute phase did not develop hyperresponsiveness, indicating that IL-13 is important for the development of the disease, at least under these conditions. In the infections caused only by male schistosomes, the cytokine profiles were similar, with the exception of higher IL-10 production in mice infected only with male schistosomes. Interestingly, mice infected with male schistosomes did not develop hyperresponsiveness, regardless of OVA sensitization. After treatment with praziquantel, the male-schistosome infected mice became susceptible to hyperresponsiveness induced by OVA. There were some differences in the cytokine profile of bronchoalveolar lavages between OVA-sensitized male-schistosome infected mice and uninfected mice, as the first group presented higher IL-4, IL-13, and IL-10 production and lower IL-5 production. Comparing these groups, the infected one also presented no lung inflammation nor peribronchial eosinophilia and lower goblet cell hyperplasia, in contrast with the noninfected group. When the authors blocked IL-10 action in the infected group, via monoclonal antibodies, the mice became susceptible to the hyperresponsiveness, with higher eosinophilia and higher IL-5 production. The resistance to the disease was also associated with B cells, as these were more present in the worm-infected mice and their partial depletion by Anti-IgMs made them susceptible to the disease.

In an approach similar to Mangan et al. [35], Mo and colleagues [36] have, instead, used* S. japonicum*. In their study, bronchoalveolar lavages of mice infected with both sexes of* S. japonicum* and mice infected with males only presented lower eosinophilia and leukocyte counts. The infection with* S. japonicum* prior to the OVA challenge reduced the parenchymal inflammation of the lungs and the production of mucus. The cytokine profile in the bronchoalveolar lavages changed as well, with lower production of IL-4 and IL-5 and a higher production of IL-10 in mice infected with either combination of worms. Lastly, they have observed less OVA-specific IgE in the serum of mice infected with both sexes of* S. japonicum* or males only.

Using similar animal models, Smits and colleagues [37] showed that chronically infected mice with* S. mansoni* presented less intense eosinophilia in the lungs and this effect depended upon the quantity of eggs used to infect the mice. These mice also presented less severe peribronchial and perivascular inflammation and fewer goblet cells in the bronchi, alongside the repression of the hyperresponsiveness. They have also observed lower expression of IL-4 and IL-13 in mediastinal lymph node cells and spleen cells of chronically infected mice, in response to OVA or SEA. Lastly, lymph node cells or spleen cells transplanted from chronically infected mice into OVA-sensitized mice diminish lung eosinophilia in the recipient mice. These effects were nullified if the recipient mice had their IL-10 cytokines blocked. Similar results were observed when the transferred cells were T CD4^+^ or CD19^+^ B cells.

Having found comparable results to previous studies, with regard to lower eosinophilia, lower inflammation in the lungs, and IL-10 increased production in mice infected with* S. mansoni*, Pacífico and colleagues [38] have shown lower levels of OVA-specific IgE in the infected mice, first report for* S. mansoni*, and, more importantly, have highlighted the importance of T regulatory cells in this context. Firstly, mice pretreated with* S. mansoni* eggs presented higher amounts of CD4^+^ CD25^+^ Foxp3^+^ T cells (T-regs) in the lungs compared to uninfected controls. These cells, however, were not the source of IL-10. Infected mice presented lower levels of CCL2, CCL3, and CCL5 in their lungs, which are chemokines related to eosinophil chemotaxis. In this study, depletion of T-regs, and not of IL-10, restored lung eosinophilia to control levels. Secondly, when either T-regs or IL-10 were depleted, cytokine and chemokine levels (IL-4 and IL-5 as cytokine indicatives and CCL2, CCL3, and CCL5 as chemokine indicatives) were restored to control levels. Lastly, only when T-regs were depleted was inflammation in the lungs of mice increased.

Amu and colleagues [39] sought to further characterize the B-reg cell populations that are involved in the suppression of the airway allergy caused by* S. Mansoni* and how they do so. They have found B cell transplantation results similar to Smits et al. [37] study. Moreover, they have found that most of the IL-10^+^ CD19^+^ cells, that is, B-regs which produce IL-10, are also CD1d^high^, CD5^+^, CD21^high^, CD23^+^, IgD^+^, and IgM^high^. Infected mice treated with anti-CD1d monoclonal antibodies became susceptible to airway allergy. Although CD1d interacts with invariant natural killer T (iNKT) cells, the authors did not observe expressive expansion of these cells. CD1d^high^ cells transplanted from infected mice into ovalbumin-primed mice made these develop less airway inflammation, less goblet cell hyperplasia, less eosinophilia, and lower values of IL-4, IL-5, IL-13, and eotaxin and higher values of IL-10 in bronchoalveolar lavages, in contrast to mice that received no cells or CD1d^low^ cells. The infection of mice also promoted the expansion of CD1d^−^ cells, which were also CD5^+^, CD21^low^, CD23^+^, IgD^+^, and IgM^low^. This population of cells, in transplant experiments similar to the previous cell population, have worsened the allergy. B-regs from infected mice transplanted into ovalbumin-primed mice promoted the expansion of T-regs in their lungs. However, when the same was done to IL-10^−/−^ mice as donors of the B-regs, this was not observed, showing that this expansion is IL-10 dependent. This was further shown by blocking IL-10 in recipient mice with anti-IL-10 antibodies. The blocking of TGF-*β* in recipient mice did not affect T-reg expansion. B-reg expansion was also possible by incubating spleen cells from healthy mice with live worms, with similar markers seen in the* in vivo* system. These cells obtained from this* ex vivo* system were also able to induce T-reg expansion in recipient mice. Lastly, B-regs transplanted into mice that had already developed airway allergies promoted reduction of eosinophilia and increase in T-regs, indicating that they have therapeutic properties.

Instead of using worms as preventive agents, Cardoso and colleagues [40] have, for the first time, used recombinant antigens from* S. mansoni* to prevent airway allergy in mice. They have used three different antigens: Sm22.6, present in the tegument of the worm in all stages of its life cycle, except for the eggs; PIII, present in SWA of adult schistosomes; and Sm29, present in the tegument of the lung stage of adult schistosomes. These antigens were produced as recombinant proteins in* Escherichia coli*. Mice treated with any of these antigens presented less lung inflammation and lower OVA-specific IgE levels and eosinophilia compared to the controls. Also, eosinophil peroxidase was lower in mice treated with either Sm22.6 or PIII. The level of IL-4 and IL-5 in bronchoalveolar lavages was lower in mice treated with either Sm22.6 or PIII and the levels of IL-10 were higher in mice treated with Sm22.6. However, the ratios of IL-10 : IL-4 were higher both in Sm22.6 treated and in PIII treated mice. The levels of IFN-*γ* were lower in mice treated with Sm29 only and no differences of TNF-*α* levels were observed. Lastly, the number of CD4^+^ Foxp3^+^ T-regs was higher in either Sm22.6 or PIII treated mice, but only mice treated with Sm22.6 presented higher levels of these cells which expressed IL-10.

Having found results similar to Amu et al. [39], van der Vlugt and colleagues [41] have also shown that the B-regs from the marginal zone of the spleen are the main B-regs in terms of IL-10 production and Foxp3^+^ T-regs enhancement in mice. They have also analysed the induction of IL-10 producing B-regs in humans infected with schistosomes. Peripheral blood mononuclear cells from Gabonese children, either positive or negative for* S. haematobium*, were screened for the presence of B-regs populations. The presence of CD1d^high^ B-regs was higher in infected children than in the uninfected. This presence lowered to comparable levels after six months of praziquantel treatment, indicating that the presence of viable worms is important for the maintenance of this B-regs population. After stimulating the B cells isolated from the blood with anti-IgM and anti-IgG antibodies, there was an increase in IL-10 from the cells isolated from the infected children, which was related to CD1d^high^ B-regs. When this stimulation was done with SEA, the production of IL-10 remained even if the children had been treated with praziquantel. Being one of the very few studies that report some form of immunomodulation in* S. haematobium*, this study shows the necessity of further characterization of the mechanisms of immunomodulation for this worm and for using it or its derivatives in the prevention or treatment of autoimmune diseases.

To better characterize how the infection stage of* S. mansoni* plays a role in the prevention of airway allergies, Layland and colleagues [42] have sensitized and challenged infected mice at different time intervals. They have observed that mice which were sensitized and challenged with OVA only after patency, that is, after female worms started to produce eggs, had the lowest amounts of lung eosinophilia, leucocyte counts, and inflammation scores, in comparison to mice that were either challenged or sensitized and challenged before the establishment of patency. In another experiment, infected mice that were treated with praziquantel before being sensitized and challenged still presented lower levels of eosinophilia and leucocyte infiltration in the lungs after 6 weeks of the treatment but had inflammation levels comparable to noninfected mice. After 12 weeks, there were no differences between noninfected mice and praziquantel-treated mice with regard to cell infiltration and inflammation in the lungs. Altogether, these results suggest the importance of having an established infection to prevent the development of the autoimmune disease. In their study, the role of regulatory (Foxp3^+^) cells was further described. They have observed higher presence of T-regs in the lymph nodes of infected mice, even if they were not exposed to OVA, but not in their lungs. A final group of mice was analysed: mice that were infected with* S. mansoni*, then had their T-regs depleted with diphtheria toxin (DT), and then were sensitized and challenged with OVA. These mice had comparable values of cell infiltration and inflammation in the lungs with uninfected, DT treated mice, which were higher than those of OVA infected or OVA uninfected mice. OVA-specific IgE levels were also higher in DT treated mice, regardless of infection presence. DT treated, infected mice, upon stimulation with SEA, also presented higher levels of IL-5 than infected mice without DT treatment. To summarise, these results indicate that even if T-regs may not be present in the lungs, they still have an important role in the prevention of the airway allergy.

Straubinger and colleagues [43] evaluated if the presence of a schistosome infection during pregnancy could help to protect offspring from autoimmune diseases, given the lower occurrence of autoimmune diseases in children that are raised in farming environments. In order to do it, female mice were mated during acute, chronic, or regulatory phases of schistosome infection and then their offspring would be sensitized and challenged with OVA. The authors have seen that different phases of schistosome infection during pregnancy can predispose differently the offspring, as offspring of mothers infected with either acute or regulatory phases had fewer lymphocytes and less eosinophilia in bronchoalveolar lavages after OVA challenges, in contrast to offspring of mothers infected in the chronic phase, which were actually worse than in the controls. The lung inflammation, goblet cell numbers, and OVA-specific IgE levels were also lower in the two previous groups than in controls and in the latter group. These differences in the disease development were not transferred via the germline, as the authors have elegantly demonstrated. They have collected oocytes from either acutely infected mice or uninfected mice; then these oocytes were fertilized* in vitro* and transferred into uninfected surrogate mothers. The resulting offspring was then sensitized and challenged with OVA. Both resulting groups presented similar leukocyte infiltration levels and OVA-specific IgE levels and similar inflammation and goblet cell formation levels. After analysing the gene expression profiles of mothers infected with different phases and uninfected mothers, through RNA microarrays, the authors have found common trends between the groups. Interestingly, IL-10, IL-5, and Foxp3, which have been used extensively in previous studies as markers of the immune response, did not have their expression profiles changed along the groups. However, there were similarities between the expression profiles of mothers infected in acute or regulatory phases, such as genes involved in serotonin and melatonin biosynthesis and mineralocorticoid and glucocorticoid biosynthesis and glycolysis. Some genes were expressed differentially only in chronically infected mothers, such as thyroid hormone metabolism and Th17-related responses. Cells isolated from placentas and deciduas from infected mice of all phases and uninfected mice were stimulated with SEA, with some differences in cytokine production from each group. High IL-4 production was found in cells from the chronically infected cells and IL-10 from the regulatory-infected cells and IFN-*γ* was found from early acutely infected cells, indicating that these differences in the expression of genes related to cytokines and to metabolic pathways in the placenta are factors of relevance in the predisposition of the offspring to develop allergies. In the end, offspring generated from acutely infected mice which lacked IFN-*γ* presented higher cell infiltrations in the lung, higher inflammation scores, and more goblet cells than offspring whose mothers were not infected or that were able to produce IFN-*γ*, showing that this cytokine is important in the protection of offspring from acutely infected mice.

A second study using isolated antigens to prevent airway hyperresponsiveness was performed, by Ren and colleagues [44]. They have used an egg protein from* S. japonicum*, named SjP40, which, as they showed, was able to induce IFN-*γ* from spleen cells isolated from infected mice. Some peptides contained within SjP40 were also able to reduce lung inflammation, mucus production, and eosinophilia. Animals treated with these peptides also presented lower levels of OVA-specific IgG1 and IgE, though they presented higher levels of OVA-specific IgG2a. Lastly, animals treated with the peptides presented lower levels of IL-4, IL-5, and IL-13 and higher levels of IFN-*γ*. Altogether, these results indicate that animals exposed to SjP40 or some of its peptides presented a shift from a Th2 to a Th1 response, in the airway allergy context.

## 5. Conclusions and Perspectives

For over two decades, since the proposition of the hygiene hypothesis, researchers from different parts of the globe have invested their efforts to understand the relationship between inflammatory and autoimmune diseases with the protective effect of helminth parasites. Many aspects and mechanisms of schistosomes and hosts interactions were unraveled enlightening the immunomodulatory role of many molecules. Most of these studies used murine models of schistosomes infection or they were based on extracts containing mixtures of molecules originated from different life stages of these parasites such as SEA, SWA, or entire eggs.

From these initial tests using purified extracts of eggs or adult* S. mansoni* worms ascended some isolated molecules as potential candidates for therapeutic use against inflammatory diseases. The glycoconjugate Lacto-N-fucopentose-III is an example of isolated molecule that retained its immunomodulatory activity preventing type II diabetes and psoriasis in animal models. The immunomodulatory effects of schistosome infection and egg-derived molecules are clear, as well as their ability to downregulate proinflammatory cytokines, upregulate anti-inflammatory cytokines, and drive a Th2 type of immune response. Despite the evident therapeutic potential of these molecules, it is difficult to presume whether a single molecule will retain the immunomodulatory effect that has been observed in animal models that used complex mixtures of schistosome-derived molecules. Biotechnological advances are offering new systems to generate recombinant proteins in eukaryotic cells, which allow higher yields of purified proteins with their posttranslation modifications such as glycosylations and acylations [93–96]. These biotechnological tools can be essential to obtain isolated molecules in their native and original form, a characteristic that is largely vital for glycoproteins and lipoproteins activity and antigenicity as previously demonstrated for helminth molecules in some publications [58, 97]. In recent years, many proteomic studies revealed the composition of peptides of soluble egg antigens (SEA), adult soluble worm antigen (SWA) preparation, cercariae, schistosomula tegument, and adult worm tegument [98–106] which, altogether, represent a vast repertoire of potential proteins to be evaluated in combinations or isolated in order to find key immunomodulatory proteins within these different and complex molecular mixtures from schistosomes.

In the coming years and decades the challenges will be centered on bioinformatics and biotechnological methods to enable the selection of target proteins from large proteomic data, to produce them in their native form in eukaryotic systems of heterologous expression to be purified and tested as isolated formulation and in different combinations in* in vitro* and* in vivo* models. Following these molecular selection and immunomodulatory screening the best proteins to be used as therapies for inflammatory and autoimmune diseases can be further explored in order to determine their three-dimensional structures and get their epitopes and domains responsible for their immunomodulatory effects, which could enable the chemical synthesis of these active sites and the obtainment of smaller molecules with the potential to replace the use of peptide molecules which are often unstable and incompatible with oral administrations.

## Figures and Tables

**Table 1:** Experimental animal models to study the role of schistosome molecules in different inflammatory and autoimmune diseases.

Disease model	Regimen	Effects	References
Arthritis (DBA/1 mice)	*S. mansoni *inf.	Prevention	Osada et al., 2009 [15]
	*S. japonicum *inf.	Prevention	Song et al., 2011 [16]

Arthritis (IL-1ra- mice)	*S. mansoni *inf.	Prevention	Osada et al., 2015 [17]

T1D (NOD mice)	SEA with or without insulin	Prevention	Maron et al., 1998 [18]
	*S. mansoni *inf. adults or eggs	Prevention	Cooke et al., 1999 [19]
	*S. mansoni *dead eggs, SEA, SWA	Prevention	Zaccone et al., 2003 [20]
	SEA	Prevention	Zaccone et al., 2009 [13]
	SEA	Prevention	Cooke et al., 2010 [21]

T2D (diet-induced mice)	*S. mansoni *inf., SEA	Amelioration	Hussaarts et al., 2015 [22]
	LNFPIII	Amelioration	Bhargava et al., 2012 [23]

MetS (apoE^−/−^ mice)	*S. mansoni *inf. adults or eggs, SEA	Amelioration	Stanley et al., 2009 [24]

MetS (LDLR^−/−^ mice)	SEA	Prevention	Wolfs et al., 2013 [25]

Graves' hyperthyroidism	*S. mansoni *inf., SEA, SWA	Prevention	Nagayama et al., 2004 [26]

Psoriasis	LNFPIII	Prevention	Atochina and Harn, 2006 [27]

IBD	SWA	Amelioration	Ruyssers et al., 2010 [28]
		Amelioration	Heylen et al., 2014 [29]

EAE	*S. mansoni *egg inf.	Amelioration	Sewell et al., 2003 [30]
	*S. mansoni *inf.	Prevention	La Flamme et al., 2003 [31]
	SEA (*S. japonicum*)	Prevention and amelioration	Zheng et al., 2008 [32]
	LNFPIII	Amelioration	Zhu et al., 2012 [33]
		Amelioration	Kuijk et al., 2012 [34]

Airway allergies	*S. mansoni *inf.	Prevention	Mangan et al., 2006 [35]
	*S. japonicum *inf.	Prevention	Mo et al., 2008 [36]
	*S. mansoni *inf.	Prevention	Smits et al., 2007 [37]
	*S. mansoni *inf.	Prevention	Pacífico et al., 2009 [38]
	*S. mansoni *inf.	Prevention and amelioration	Amu et al., 2010 [39]
	Sm22.6, PIII, Sm29	Prevention	Cardoso et al., 2010 [40]
	*S. mansoni *or *S. haematobium *inf.	Prevention	van der Vlugt et al., 2012 [41]
	*S. mansoni *inf.	Prevention	Layland et al., 2013 [42]
	*S. mansoni *inf. in mother mice	Prevention or Exacerbation	Straubinger et al., 2013 [43]
	SjP40	Prevention	Ren et al., 2016 [44]

IL-1ra: interleukin 1 receptor antagonist; T1D: type 1 diabetes; NOD: nonobese diabetic; SEA: soluble egg antigens; SWA: soluble worm antigens; LNFPIII: Lacto-N-fucopentose-III; T2D: type 2 diabetes; MetS: metabolic syndrome; apoE^−/−^: apolipoprotein E deficient mice; LDLR^−/−^: low density lipoprotein receptor deficient mice; IBD: inflammatory bowel disease; EAE: experimental autoimmune encephalomyelitis; Sm22.6: *S. mansoni* protein with 22.6 kDa, purified as recombinant protein; Sm29: *S. mansoni* protein with 29 kDa, purified as recombinant protein; and PIII: antigen from SWA, purified as recombinant protein.

## References

[1] McSorley H. J., Maizels R. M. (2012). Helminth infections and host immune regulation. *Clinical Microbiology Reviews*.

[2] McSorley H. J., Hewitson J. P., Maizels R. M. (2013). Immunomodulation by helminth parasites: defining mechanisms and mediators. *International Journal for Parasitology*.

[3] Strachan D. P. (1989). Hay fever, hygiene, and household size. *British Medical Journal*.

[4] Bashi T., Bizzaro G., Ben-Ami Shor D., Blank M., Shoenfeld Y. (2015). The mechanisms behind helminth's immunomodulation in autoimmunity. *Autoimmunity Reviews*.

[5] Daniłowicz-Luebert E., O'Regan N. L., Steinfelder S., Hartmann S. (2011). Modulation of specific and allergy-related immune responses by helminths. *Journal of Biomedicine and Biotechnology*.

[6] Erb K. J. (2009). Can helminths or helminth-derived products be used in humans to prevent or treat allergic diseases?. *Trends in Immunology*.

[7] Cooke A. (2012). Parasitic worms and inflammatory disease. *Current Opinion in Rheumatology*.

[8] Guigas B., Molofsky A. B. (2015). A worm of one's own: how helminths modulate host adipose tissue function and metabolism. *Trends in Parasitology*.

[9] Wiria A. E., Djuardi Y., Supali T., Sartono E., Yazdanbakhsh M. (2012). Helminth infection in populations undergoing epidemiological transition: a friend or foe?. *Seminars in Immunopathology*.

[10] Allen J. E., Maizels R. M. (2011). Diversity and dialogue in immunity to helminths. *Nature Reviews Immunology*.

[11] Cooke A. (2009). Review series on helminths, immune modulation and the hygiene hypothesis: how might infection modulate the onset of type 1 diabetes?. *Immunology*.

[12] Harnett W., Harnett M. M. (2010). Helminth-derived immunomodulators: can understanding the worm produce the pill?. *Nature Reviews Immunology*.

[13] Zaccone P., Burton O., Miller N., Jones F. M., Dunne D. W., Cooke A. (2009). Schistosoma mansoni egg antigens induce Treg that participate in diabetes prevention in NOD mice. *European Journal of Immunology*.

[14] Zaccone P., Burton O. T., Gibbs S. (2010). Immune modulation by *Schistosoma mansoni* antigens in NOD mice: effects on both innate and adaptive immune systems. *Journal of Biomedicine and Biotechnology*.

[15] Osada Y., Shimizu S., Kumagai T., Yamada S., Kanazawa T. (2009). *Schistosoma mansoni* infection reduces severity of collagen-induced arthritis via down-regulation of pro-inflammatory mediators. *International Journal for Parasitology*.

[16] Song X., Shen J., Wen H. (2011). Impact of Schistosoma japonicum infection on collagen-induced arthritis in DBA/1 mice: a murine model of human rheumatoid arthritis. *PLoS ONE*.

[17] Osada Y., Yamada S., Nakae S., Sudo K., Kanazawa T. (2014). Reciprocal effects of *Schistosoma mansoni* infection on spontaneous autoimmune arthritis in IL-1 receptor antagonist-deficient mice. *Parasitology International*.

[18] Maron R., Palanivel V., Weiner H. L., Harn D. A. (1998). Oral administration of schistosome egg antigens and insulin B-chain generates and enhances Th2-type responses in NOD mice. *Clinical Immunology and Immunopathology*.

[19] Cooke A., Tonks P., Jones F. M. (1999). Infection with *Schistosoma mansoni* prevents insulin dependent diabetes mellitus in non-obese diabetic mice. *Parasite Immunology*.

[20] Zaccone P., Feheérvári Z., Jones F. M. (2003). *Schistosoma mansoni* antigens modulate the activity of the innate immune response and prevent onset of type 1 diabetes. *European Journal of Immunology*.

[21] Cooke A., Zaccone P., Burton O. T. (2010). Immune modulation by schistosoma mansoni antigens in NOD mice: effects on both innate and adaptive immune systems. *Journal of Biomedicine and Biotechnology*.

[22] Hussaarts L., García-Tardón N., Van Beek L. (2015). Chronic helminth infection and helminth-derived egg antigens promote adipose tissue M2 macrophages and improve insulin sensitivity in obese mice. *The FASEB Journal*.

[23] Bhargava P., Li C., Stanya K. J. (2012). Immunomodulatory glycan LNFPIII alleviates hepatosteatosis and insulin resistance through direct and indirect control of metabolic pathways. *Nature Medicine*.

[24] Stanley R. G., Jackson C. L., Griffiths K., Doenhoff M. J. (2009). Effects of Schistosoma mansoni worms and eggs on circulating cholesterol and liver lipids in mice. *Atherosclerosis*.

[25] Wolfs I. M., Stoger J. L., Goossens P. (2014). Reprogramming macrophages to an anti-inflammatory phenotype by helminth antigens reduces murine atherosclerosis. *The FASEB Journal*.

[26] Nagayama Y., Watanabe K., Niwa M., McLachlan S. M., Rapoport B. (2004). Schistosoma mansoni and *α*-galactosylceramide: prophylactic effect of Th1 immune suppression in a mouse model of Graves' hyperthyroidism. *The Journal of Immunology*.

[27] Atochina O., Harn D. (2006). Prevention of psoriasis-like lesions development in fsn/fsn mice by helminth glycans. *Experimental Dermatology*.

[28] Ruyssers N. E., De Winter B. Y., De Man J. G. (2010). *Schistosoma mansoni* proteins attenuate gastrointestinal motility disturbances during experimental colitis in mice. *World Journal of Gastroenterology*.

[29] Heylen M., Ruyssers N. E., De Man J. G. (2014). Worm proteins of *Schistosoma mansoni* reduce the severity of experimental chronic colitis in mice by suppressing colonic proinflammatory immune responses. *PLoS ONE*.

[30] Sewell D., Qing Z., Reinke E. (2003). Immunomodulation of experimental autoimmune encephalomyelitis by helminth ova immunization. *International Immunology*.

[31] La Flamme A. C., Canagasabey K., Harvie M., Bäckström B. T. (2004). Schistosomiasis protects against multiple sclerosis. *Memorias do Instituto Oswaldo Cruz*.

[32] Zheng X., Hu X., Zhou G. (2008). Soluble egg antigen from Schistosoma japonicum modulates the progression of chronic progressive experimental autoimmune encephalomyelitis via Th2-shift response. *Journal of Neuroimmunology*.

[33] Zhu B., Trikudanathan S., Zozulya A. L. (2012). Immune modulation by Lacto-N-fucopentaose III in experimental autoimmune encephalomyelitis. *Clinical Immunology*.

[34] Kuijk L. M., Klaver E. J., Kooij G. (2012). Soluble helminth products suppress clinical signs in murine experimental autoimmune encephalomyelitis and differentially modulate human dendritic cell activation. *Molecular Immunology*.

[35] Mangan N. E., van Rooijen N., McKenzie A. N. J., Fallon P. G. (2006). Helminth-modified pulmonary immune response protects mice from allergen-induced airway hyperresponsiveness. *The Journal of Immunology*.

[36] Mo H.-M., Lei J.-H., Jiang Z.-W. (2008). Schistosoma japonicum infection modulates the development of allergen-induced airway inflammation in mice. *Parasitology Research*.

[37] Smits H. H., Hammad H., van N. M. (2007). Protective effect of *Schistosoma mansoni* infection on allergic airway inflammation depends on the intensity and chronicity of infection. *The Journal of Allergy and Clinical Immunology*.

[38] Pacífico L. G. G., Marinho F. A. V., Fonseca C. T. (2009). Schistosoma mansoni antigens modulate experimental allergic asthma in a murine model: a major role for CD4^+^ CD25^+^ Foxp3^+^ T cells independent of interleukin-10. *Infection and Immunity*.

[39] Amu S., Saunders S. P., Kronenberg M., Mangan N. E., Atzberger A., Fallon P. G. (2010). Regulatory B cells prevent and reverse allergic airway inflammation via FoxP3-positive T regulatory cells in a murine model. *Journal of Allergy and Clinical Immunology*.

[40] Cardoso L. S., Oliveira S. C., Góes A. M. (2010). Schistosoma mansoni antigens modulate the allergic response in a murine model of ovalbumin-induced airway inflammation. *Clinical & Experimental Immunology*.

[41] van der Vlugt L. E. P. M., Labuda L. A., Ozir-Fazalalikhan A. (2012). Schistosomes induce regulatory features in human and mouse CD1d^hi^ B cells: inhibition of allergic inflammation by IL-10 and regulatory T cells. *PLoS ONE*.

[42] Layland L. E., Straubinger K., Ritter M. (2013). *Schistosoma mansoni*-mediated suppression of allergic airway inflammation requires patency and Foxp3^+^ Treg cells. *PLoS Neglected Tropical Diseases*.

[43] Straubinger K., Paul S., Prazeres da Costa O. (2014). Maternal immune response to helminth infection during pregnancy determines offspring susceptibility to allergic airway inflammation. *Journal of Allergy and Clinical Immunology*.

[44] Ren J., Hu L., Yang J. (2016). Novel T-cell epitopes on *Schistosoma japonicum* SjP40 protein and their preventive effect on allergic asthma in mice. *European Journal of Immunology*.

[45] Fleming J. O., Weinstock J. V. (2015). Clinical trials of helminth therapy in autoimmune diseases: rationale and findings. *Parasite Immunology*.

[46] Evans H., Mitre E. (2015). Worms as therapeutic agents for allergy and asthma: understanding why benefits in animal studies have not translated into clinical success. *Journal of Allergy and Clinical Immunology*.

[47] Pritchard D. I., Blount D. G., Schmid-Grendelmeier P., Till S. J. (2012). Parasitic worm therapy for allergy: is this incongruous or avant-garde medicine?. *Clinical and Experimental Allergy*.

[48] Nampijja M., Webb E. L., Kaweesa J. (2015). The Lake Victoria island intervention study on worms and allergy-related diseases (LaVIISWA): study protocol for a randomised controlled trial. *Trials*.

[49] Cohen F. E., Gregoret L. M., Amiri P., Aldape K., Railey J., McKerrow J. H. (1991). Arresting tissue invasion of a parasite by protease inhibitors chosen with the aid of computer modeling. *Biochemistry*.

[50] Curwen R. S., Ashton P. D., Sundaralingam S., Wilson R. A. (2006). Identification of novel proteases and immunomodulators in the secretions of Schistosome cercariae that facilitate host entry. *Molecular and Cellular Proteomics*.

[51] Keene W. E., Jeong K. H., McKerrow J. H., Werb Z. (1983). Degradation of extracellular matrix by larvae of *Schistosoma mansoni*. II. Degradation by newly transformed and developing schistosomula. *Laboratory Investigation*.

[52] Sanin D. E., Mountford A. P. (2015). Sm16, a major component of Schistosoma mansoni cercarial excretory/secretory products, prevents macrophage classical activation and delays antigen processing. *Parasites and Vectors*.

[53] Brännström K., Sellin M. E., Holmfeldt P., Brattsand M., Gullberg M. (2009). The *Schistosoma mansoni* protein Sm16/SmSLP/SmSPO-1 assembles into a nine-subunit oligomer with potential to inhibit toll-like receptor signaling. *Infection and Immunity*.

[54] Jenkins S. J., Hewitson J. P., Jenkins G. R., Mountford A. P. (2005). Modulation of the host's immune response by schistosome larvae. *Parasite Immunology*.

[55] Richter D., Incani R. I., Harn D. A. (1996). Lacto-N- fucopentaose III (Lewis) a target of the antibody response in mice vaccinated with irradiated cercariae of *Schistosoma mansoni*. *Infection and Immunity*.

[56] Okano M., Satoskar A. R., Nishizaki K., Harn D. A. (2001). Lacto-N-fucopentaose III found on *Schitosoma mansoni* egg antigens functions as adjuvant for proteins by inducing Th2-type response1. *Journal of Immunology*.

[57] van Liempt E., van Vliet S. J., Engering A. (2007). *Schistosoma mansoni* soluble egg antigens are internalized by human dendritic cells through multiple C-type lectins and suppress TLR-induced dendritic cell activation. *Molecular Immunology*.

[58] Kuijk L. M., van Die I. (2010). Worms to the rescue: can worm glycans protect from autoimmune diseases?. *IUBMB Life*.

[59] van Die I., van Vliet S. J., Nyame A. K. (2003). The dendritic cell-specific C-type lectin DC-SIGN is a receptor for *Schistosoma mansoni* egg antigens and recognizes the glycan antigen Lewis x. *Glycobiology*.

[60] Everts B., Hussaarts L., Driessen N. N. (2012). Schistosome-derived omega-1 drives Th2 polarization by suppressing protein synthesis following internalization by the mannose receptor. *The Journal of Experimental Medicine*.

[61] Liao Q., Yuan X., Xiao H. (2011). Identifying schistosoma japonicum excretory/secretory proteins and their interactions with host immune system. *PLoS ONE*.

[62] Meevissen M. H. J., Yazdanbakhsh M., Hokke C. H. (2012). Schistosoma mansoni egg glycoproteins and C-type lectins of host immune cells: molecular partners that shape immune responses. *Experimental Parasitology*.

[63] Pearce E. J., MacDonald A. S. (2002). The immunobiology of schistosomiasis. *Nature Reviews Immunology*.

[64] Sotillo J., Pearson M., Becker L., Mulvenna J., Loukas A. (2015). A quantitative proteomic analysis of the tegumental proteins from *Schistosoma mansoni* schistosomula reveals novel potential therapeutic targets. *International Journal for Parasitology*.

[65] Wu C., Hou N., Piao X. (2015). Non-immune immunoglobulins shield *Schistosoma japonicum* from host immunorecognition. *Scientific Reports*.

[66] Loukas A., Jones M. K., King L. T., Brindley P. J., McManus D. P. (2001). Receptor for Fc on the surfaces of schistosomes. *Infection and Immunity*.

[67] Deng J., Gold D., LoVerde P. T., Fishelson Z. (2003). Inhibition of the complement membrane attack complex by *Schistosoma mansoni* paramyosin. *Infection and Immunity*.

[68] Mebius M. M., van Genderen P. J. J., Urbanus R. T., Tielens A. G. M., De Groot P. G., van Hellemond J. J. (2013). Interference with the host haemostatic system by schistosomes. *PLoS Pathogens*.

[69] Wu Y. P., Lenting P. J., Tielens A. G. M., De Groot P. G., Van Hellemond J. J. (2007). Differential platelet adhesion to distinct life-cycle stages of the parasitic helminth *Schistosoma mansoni*. *Journal of Thrombosis and Haemostasis*.

[70] Figueiredo B. C., Da'dara A. A., Oliveira S. C., Skelly P. J. (2015). Schistosomes enhance plasminogen activation: the role of tegumental enolase. *PLoS Pathogens*.

[71] Da'dara A. A., Bhardwaj R., Ali Y. B. M., Skelly P. J. (2014). Schistosome tegumental ecto-apyrase (SmATPDase1) degrades exogenous pro-inflammatory and pro-thrombotic nucleotides. *PeerJ*.

[72] Da'Dara A. A., Skelly P. J. (2014). Schistosomes versus platelets. *Thrombosis Research*.

[73] Lin Y.-L., He S. (2006). Sm22.6 antigen is an inhibitor to human thrombin. *Molecular and Biochemical Parasitology*.

[74] Steinfelder S., Andersen J. F., Cannons J. L. (2009). The major component in schistosome eggs responsible for conditioning dendritic cells for Th2 polarization is a T2 ribonuclease (omega-1). *The Journal of Experimental Medicine*.

[75] Schramm G., Mohrs K., Wodrich M. (2007). Cutting edge: IPSE/alpha-1, a glycoprotein from *Schistosoma mansoni* eggs, induces IgE-dependent, antigen-independent IL-4 production by murine basophils in vivo. *The Journal of Immunology*.

[76] Wu C., Chen Q., Fang Y. (2014). Schistosoma japonicum Egg specific protein SjE16.7 recruits neutrophils and induces inflammatory hepatic granuloma initiation. *PLoS Neglected Tropical Diseases*.

[77] He Y., Li J., Zhuang W. (2010). The inhibitory effect against collagen-induced arthritis by *Schistosoma japonicum* infection is infection stage-dependent. *BMC Immunology*.

[78] Atkinson M. A., Eisenbarth G. S., Michels A. W. (2014). Type 1 diabetes. *The Lancet*.

[79] Lee B.-C., Lee J. (2014). Cellular and molecular players in adipose tissue inflammation in the development of obesity-induced insulin resistance. *Biochimica et Biophysica Acta—Molecular Basis of Disease*.

[80] Chen Y., Lu J., Huang Y. (2013). Association of previous schistosome infection with diabetes and metabolic syndrome: a cross-sectional study in rural China. *The Journal of Clinical Endocrinology & Metabolism*.

[81] Hotamisligil G. S., Shargill N. S., Spiegelman B. M. (1993). Adipose expression of tumor necrosis factor-*α*: direct role in obesity-linked insulin resistance. *Science*.

[82] Uysal K. T., Wiesbrock S. M., Marino M. W., Hotamisligil G. S. (1997). Protection from obesity-induced insulin resistance in mice lacking TNF-*α* function. *Nature*.

[83] Pradhan A. D., Manson J. E., Rifai N., Buring J. E., Ridker P. M. (2001). C-reactive protein, interleukin 6, and risk of developing type 2 diabetes mellitus. *The Journal of the American Medical Association*.

[84] Lumeng C. N., DeYoung S. M., Bodzin J. L., Saltiel A. R. (2007). Increased inflammatory properties of adipose tissue macrophages recruited during diet-induced obesity. *Diabetes*.

[85] Lumeng C. N., Bodzin J. L., Saltiel A. R. (2007). Obesity induces a phenotypic switch in adipose tissue macrophage polarization. *Journal of Clinical Investigation*.

[86] Xu H., Barnes G. T., Yang Q. (2003). Chronic inflammation in fat plays a crucial role in the development of obesity-related insulin resistance. *The Journal of Clinical Investigation*.

[87] Véniant M. M., Withycombe S., Young S. G. (2001). Lipoprotein size and atherosclerosis susceptibility in Apoe^−/−^ and Ldlr^−/−^ mice. *Arteriosclerosis, Thrombosis, and Vascular Biology*.

[88] Chachu K. A., Osterman M. T. (2016). How to diagnose and treat IBD mimics in the refractory IBD patient who does not have IBD. *Inflammatory Bowel Diseases*.

[89] Yamamoto-Furusho J. K., Podolsky D. K. (2007). Innate immunity in inflammatory bowel disease. *World Journal of Gastroenterology*.

[90] Hasby E. A., Hasby Saad M. A., Shohieb Z., El Noby K. (2015). FoxP3+ T regulatory cells and immunomodulation after Schistosoma mansoni egg antigen immunization in experimental model of inflammatory bowel disease. *Cellular Immunology*.

[91] Thomas P. G., Harn D. A. (2004). Immune biasing by helminth glycans. *Cellular Microbiology*.

[92] Elliott D. E., Summers R. W., Weinstock J. V. (2005). Helminths and the modulation of mucosal inflammation. *Current Opinion in Gastroenterology*.

[93] Breitling R., Klingner S., Callewaert N. (2002). Non-pathogenic trypanosomatid protozoa as a platform for protein research and production. *Protein Expression and Purification*.

[94] Dinculescu A., Hugh McDowell J., Amici S. A. (2002). Insertional mutagenesis and immunochemical analysis of visual arrestin interaction with rhodopsin. *The Journal of Biological Chemistry*.

[95] Guo S., Skala W., Magdolen V. (2016). A single glycan at the 99-loop of human kallikrein-related peptidase 2 regulates activation and enzymatic activity. *The Journal of Biological Chemistry*.

[96] Rooney B., Piening T., Büscher P., Rogé S., Smales C. M. (2015). Expression of *Trypanosoma brucei gambiense* antigens in *Leishmania tarentolae*. potential for use in rapid serodiagnostic tests (RDTs). *PLoS Neglected Tropical Diseases*.

[97] Sotillo J., Cortés A., Muñoz-Antoli C., Fried B., Esteban J. G., Toledo R. (2014). The effect of glycosylation of antigens on the antibody responses against *Echinostoma caproni* (Trematoda: Echinostomatidae). *Parasitology*.

[98] Bernardo C., Cunha M. C., Santos J. H. (2016). Insight into the molecular basis of *Schistosoma haematobium*-induced bladder cancer through urine proteomics. *Tumor Biology*.

[99] Boamah D., Kikuchi M., Huy N. T. (2012). Immunoproteomics identification of major IgE and IgG4 reactive schistosoma japonicum adult worm antigens using chronically infected human plasma. *Tropical Medicine and Health*.

[100] Cao X., Fu Z., Zhang M. (2016). iTRAQ-based comparative proteomic analysis of excretory-secretory proteins of schistosomula and adult worms of *Schistosoma japonicum*. *Journal of Proteomics*.

[101] Dorsey C. H., Stirewalt M. A. (1971). *Schistosoma mansoni*: fine structure of cercarial acetabular glands. *Experimental Parasitology*.

[102] Liu M., Ju C., Du X.-F. (2015). Proteomic analysis on cercariae and schistosomula in reference to potential proteases involved in host invasion of Schistosoma japonicum larvae. *Journal of Proteome Research*.

[103] Ludolf F., Patrocínio P. R., Corrêa-Oliveira R. (2014). Serological screening of the *Schistosoma mansoni* adult worm proteome. *PLoS Neglected Tropical Diseases*.

[104] Mathieson W., Wilson R. A. (2010). A comparative proteomic study of the undeveloped and developed *Schistosoma mansoni* egg and its contents: the miracidium, hatch fluid and secretions. *International Journal for Parasitology*.

[105] Skelly P. J., Da'dara A. A., Li X. H., Castro-Borges W., Wilson R. A. (2014). Schistosome feeding and regurgitation. *PLOS Pathogens*.

[106] Wilson R. A. (2012). Proteomics at the schistosome-mammalian host interface: any prospects for diagnostics or vaccines?. *Parasitology*.

